# Firearm-Related Injury Hospital Admissions During the COVID-19 Pandemic

**DOI:** 10.1001/jamanetworkopen.2024.56234

**Published:** 2025-01-27

**Authors:** Raymond A. Jean, Aayushi Sinha, Sarah D. Diaz, Katerina Jou, Staci T. Aubry, Mark R. Hemmila, Andrew M. Ibrahim

**Affiliations:** 1 Division of Acute Care Surgery, Department of Surgery, University of Michigan, Ann Arbor; 2 Center for Healthcare Outcomes and Policy, University of Michigan, Ann Arbor; 3 Department of Surgery, North Shore University Hospital, Manhasset, New York

## Abstract

This cohort study examines expected vs observed rates of firearm-related injury hospitalizations in the US during the COVID-19 pandemic.

## Introduction

While attention has been given to the impact of the initial COVID-19 lockdown on admissions for acute medical conditions, the pandemic’s overall effect on trauma-related conditions is much less clear. Unlike medical conditions such as stroke and myocardial infarction, which appear to have declined,^1^ rates of trauma-related hospitalizations may have increased as a result of increased gun ownership and stay-at-home orders.^2,3,4^ A better understanding of firearm-related hospitalization rates during COVID-19 would be timely, as the US Surgeon General has renewed calls to address firearm violence as a public health crisis.^5^

## Methods

We performed a longitudinal cohort study using the Healthcare Cost and Utilization Project’s National Inpatient Sample (NIS). We evaluated weighted discharges with a primary or secondary *International Statistical Classification of Diseases and Related Health Problems, Tenth Revision* diagnosis code for firearm-related injury (codes X72-X74, X93-X95, Y22-Y24, and Y35.0X) from 2015 to 2021. The STROBE reporting guidelines were followed as appropriate. This study involves secondary use of an existing database and was, therefore, granted exempt status as not regulated by the University of Michigan Medical School institutional review board; informed consent was waived for the same reason.

We generated seasonal autoregressive integrated moving average (SARIMA) and compared forecasted curves based on prepandemic trends. Seasonal trends were plotted with 95% CIs surrounding forecasted admissions. Generalized linear Poisson regression was used to estimate 2020 and 2021 annual discharges for demographic categories, including sex, age, race and ethnicity, urban vs rural designation, and insurance status based on prepandemic trends, and we quantified changes by using observed-to-expected (O:E) ratios. Race and ethnicity are collapsed into a single variable in the NIS, and were defined by the participating hospitals. Bootstrapping with replacement was used to calculate 95% CIs for O:E ratios. To identify associations with gun purchases, we calculated Pearson cross-correlation (ρ) between admissions and previously published estimates of national firearm-purchases.^6^ All statistical analyses were performed using Python software version 3.11.4 (Python Software Foundation).

## Results

We identified 159 215 weighted hospitalizations for firearm-related injury between 2015 and 2021. From 2015 to 2019, there was a steady decline in discharges that was forecasted to continue by the SARIMA model (Figure). Our models estimated 39 212 hospitalizations over 2020 to 2021, but the actual observed number was 52 480 (O:E ratio, 1.34; 95% CI, 1.19-1.48) (Table). Compared with historical trends, there were disproportionate increases in hospitalizations among patients younger than 18 years (O:E ratio, 1.44; 95% CI, 1.01-1.85), Medicaid-enrolled patients (O:E ratio, 1.46; 95% CI, 1.18-1.69), and Black patients (O:E ratio, 1.41; 95% CI, 1.13-1.63).

**Figure.**
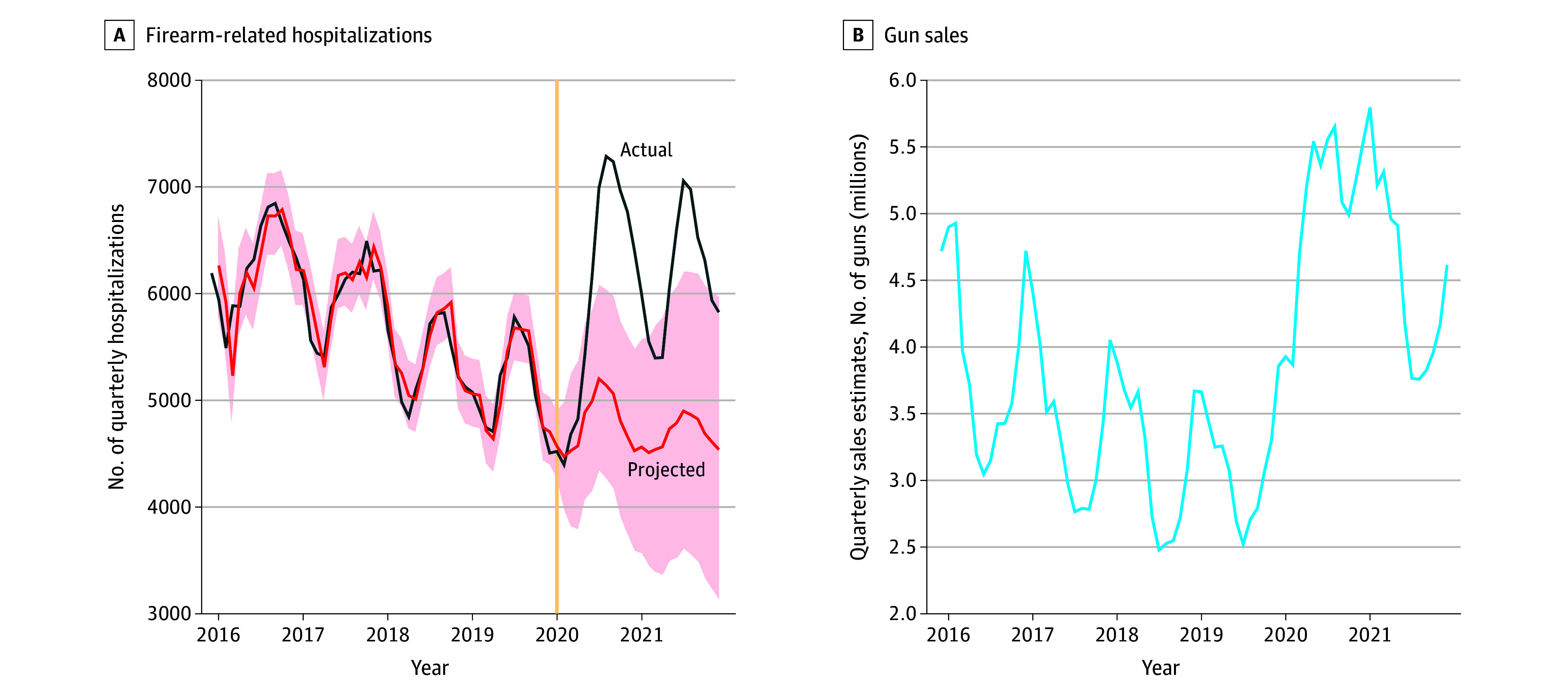
Actual vs Projected Quarterly Discharges for Firearm-Related Injuries, Accounting for Seasonal Variation (2015-2021) Data are from the National Inpatient Sample, quarter 4, 2015 to 2021, Healthcare Cost Utilization Project (HCUP; https://hcup-us.ahrq.gov/nisoverview.jsp), and estimated gun sales data are from the nonprofit organization Brady: United Against Gun Violence.^6^ The model was optimized by Akaike Information Criterion to a seasonal autoregressive integrated moving average (SARIMA) (1,1,3) (1,0,0,12), denoting the order of autoregression, differencing, and moving average components for both the nonseasonal and seasonal parts of the model. Appropriate annual weights from HCUP were applied to determine nationally representative estimates. Cross-correlation between gun purchase estimates and firearm-related admissions suggests a significant association with a 6-month lag between purchases and admission (ρ = 0.625). To minimize coding biases between *International Classification of Diseases, Ninth Revision* and *International Statistical Classification of Diseases and Related Health Problems, Tenth Revision*, the study period began in the fourth quarter of 2015. Shaded areas in panel A denote 95% CIs for the SARIMA-estimated discharges.

**Table.** Observed vs Expected Frequencies of Firearm-Related Hospital Admissions by Patient Demographics, 2020-2021^a^

Demographic characteristic	No. of hospital admissions		
	Expected (based on 2015-2019 trends)^b^	Actual observed	Observed-to-expected ratio (95% CI)
Total	39 212	52 480	1.34 (1.19-1.48)
Race			
Asian or Pacific Islander	398	505	1.27 (1.08-1.59)
Black	19 195	27 015	1.41 (1.13-1.63)
Hispanic	5884	7610	1.29 (1.00-1.52)
Missing	1669	2315	1.39 (1.19-1.56)
White	10 220	12 505	1.22 (1.05-1.49)
Other^c^	1846	2530	1.37 (1.17-1.55)
Sex			
Female	5040	7020	1.39 (1.22-1.57)
Male	34 172	45 460	1.33 (1.16-1.49)
Age category, y			
<18	2988	4305	1.44 (1.01-1.85)
18-35	22 292	30 185	1.35 (1.10-1.58)
36-54	9721	13 025	1.34 (1.24-1.44)
≥55	4211	4965	1.18 (0.86-1.64)
Primary payer			
Medicare	3842	4015	1.05 (0.68-1.65)
Medicaid	18 170	26 520	1.46 (1.18-1.69)
Private insurance	7347	9735	1.33 (1.19-1.46)
Self-pay	6722	9035	1.34 (1.06-1.58)
No charge	530	475	0.90 (0.70-1.11)
Other	2601	2700	1.04 (0.91-1.18)
Urban or rural			
Urban	35 092	47 095	1.34 (1.18-1.50)
Rural	4120	5385	1.31 (1.12-1.54)

^a^ Data are from the National Inpatient Sample, quarter 4, 2015 to 2021, Healthcare Cost Utilization Project (https://hcup-us.ahrq.gov/nisoverview.jsp).

^b^ Expected values were generated using Poisson regression trained using quarter 4 2015 to 2019 trends.

^c^ Other race includes American Indian, Native American, multiple races, or any other race not otherwise specified.

## Discussion

Our cohort study evaluating hospitalizations for firearm-related injuries after the COVID-19 pandemic has 2 principal findings. From 2020 to 2021, there was a 34% increase in firearm-related hospitalizations, consistent with an additional 13 268 admissions. Second, the increase in firearm-related hospitalization disproportionately affected children, patients enrolled in Medicaid, and Black individuals. Taken together, our findings raise new concerns about the sustained increase in firearm-related injury that disproportionately affected younger and minoritized Americans.

There are several limitations of this study. First, our data are observational and cannot directly correlate the increase in firearm sales to owners and those who are responsible for the injuries. Next, our analysis does not capture prehospital firearm deaths or injuries that did not result in a hospital admission. Similarly, the NIS does not include granular location or admission dates, so we are limited in our ability to identify events associated with specific causal increases in admissions, such as state gun laws, location-specific stay-at-home orders, or violence secondary to social unrest. Despite these limitations, these results confirm a pervasive and troubling phenomenon and suggest the need for urgent policy efforts to mitigate gun harm.^5^

## Notes

Supplement.
Data Sharing Statement
