# Supplementary material for: Firearm-Related Injury Hospital Admissions During the COVID-19 Pandemic
Source: JAMA Netw Open. 2025 Jan 27;8(1):e2456234. doi: 10.1001/jamanetworkopen.2024.56234 (PMC11774088; doi:10.1001/jamanetworkopen.2024.56234)
Supplement: Supplement. — Data Sharing Statement [file jamanetwopen-e2456234-s001.pdf]

Data Sharing Statement

Jean. Firearm-Related Injury Hospital Admissions During the COVID-19 Pandemic. *JAMA Netw Open*. Published January 27, 2025. doi:10.1001/jamanetworkopen.2024.56234

Data
Data available: No
